# Books, Good and Otherwise

**Published:** 1886-12-11

**Authors:** 


### Books, Good and Otherwise.

*Pharmacopœia of the London Lock Hospital.* London: Adlard.

We have received a copy of this little work. It is well selected, well arranged, brief, comprehensive and useful. We are glad to see that Australian beef forms a part of the dietary at the Lock. Among the ferruginous preparations we fail to find the famous Blaud's pills. Do the physicians at the Lock consider that these pills should only be administered to women? Or do they not prescribe them at all for their hospital patients? There is a rather important misprint on p. 22, which it will be well to correct in another edition.

---

*Medical Work and Duty.* An address by George Johnson, M.D., F.R.S. London: The British Medical Association.

Dr. George Johnson performed a great service for the King's College students when he gave them this address at the opening of the current session. He has added to their obligations, and placed other persons under similar ones, by its publication. The address is in every sense worthy not only of that but of any occasion. It is thoughtful, benevolent, sympathetic, learned, and simple. If every student will commit it to memory at the beginning of his career, and carry it out in practice during his career, he may be assured not only that he will pass all examinations as they fall due, but that when he has finished he will find himself possessed both of sound professional attainments and of sterling personal merit. To the general reader the remarks on special hospitals and on specialisation in practice will be found decidedly interesting. Dr. Johnson knows well, not only where specialism should begin, but also where it should end. There are other persons who might be the better for the same knowledge.

